# Enhancing Imaging Performance and Resolution in Magneto‐Acoustic Electrical Tomography With Magnetic Field Measurements (MAET‐MI) Using Figure‐of‐Eight and High‐Quality Factor Circular Coils

**DOI:** 10.1002/cnm.70063

**Published:** 2025-07-09

**Authors:** Ahmet Önder Tetik, Nevzat Güneri Gençer

**Affiliations:** ^1^ Electrical and Electronics Engineering Department Middle East Technical University Ankara Türkiye

**Keywords:** coil, delay‐and‐sum method, magneto‐acousto‐electrical tomography, ultrasound imaging, wiener filter

## Abstract

Magneto‐acousto‐electrical tomography with magnetic field measurement technique (MAET‐MI) is a hybrid imaging method that brings high spatial resolution of ultrasound imaging in electrical impedance tomography. This study investigates the impact of the quality factor of circular and figure‐of‐eight coils on the imaging performance of MAET‐MI. Induced MAET signals on the circular coil are accurately obtained by modeling a circuit representation of an air‐cored circular coil and deriving its transfer function through impedance measurements. The study demonstrates a significant improvement in signal‐to‐noise ratio (SNR) using high‐quality factor coils compared to unity quality factor coils. Additionally, a 16‐element linear phased array (LPA) ultrasound transducer, an air core circular coil, and a figure‐of‐eight coil are numerically modeled to obtain sector scan images of two‐dimensional conductivity distributions. Point spread function (PSF) is characterized, and the lateral resolution of sector scan conductivity images is enhanced through two‐dimensional deconvolution with PSF. The combined use of circular and figure‐of‐eight coils provides comprehensive imaging coverage. Notably, this research presents a practical method for estimating both circular and figure‐of‐eight coils' transfer functions, achieving 12.9 dB SNR improvement with high‐quality factor coils. A simplified breast model is rotated 16 steps, and sector scan conductive boundary images are reconstructed for both coils. A two‐dimensional image of a breast model is obtained by combining images from two different coils. These findings offer significant advancements in MAET‐MI imaging, particularly in low SNR environments.

## Graphical abstract

This study demonstrates that high‐quality‐factor coils significantly improve the SNR in MAET‐MI imaging. A practical method is proposed to estimate the circular and figure‐of‐eight coils' transfer function and reconstruct induced signals. Additionally, spatial resolution of 2D reconstructed conductive boundary image is enhanced using 2D deconvolution with the point spread function.

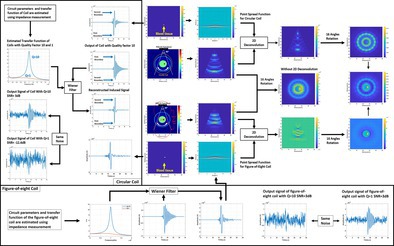

## 1 Introduction

Magneto‐acousto‐electrical tomography is a hybrid imaging method that combines the high spatial resolution of ultrasound imaging in electrical impedance tomography imaging [1]. In this method, the conductive body is held in a static magnetic field. Acoustic waves applied by the ultrasound transducer vibrate boundaries between different conductive tissues. Electrical currents are induced by Lorentz forces generated by the vibration of conductive tissues in the static magnetic field. These currents are measured with electrodes in MAET [1, 2, 3, 4, 5, 6, 7, 8, 9, 10, 11, 12, 13, 14, 15, 16, 17]. MAET with magnetic field measurement technique (MAET‐MI) uses coils as receiving sensors. In Wen et al. [1], the idea of using coils is first mentioned to measure the electric currents generated by the Lorentz force. By measuring this voltage, the conductivity map of the object is reconstructed. Previous studies have explored different approaches for MAET with magnetic field measurements [18, 19, 20, 21, 22]. Guo et al. [18] used a single‐element piston transducer to apply a pressure wave to the phantom, and MAET signals were received with a circular coil [18]. Levenberg‐Marquart (LM) algorithm was used to reconstruct conductivity distribution. In Zengin et al. [19], a linear phased array (LPA) transducer and coils sensitive to X and Y directions were employed to obtain MAET signals for each steering angle, and Truncated Singular Value Decomposition (TSVD) was utilized for conductivity map reconstruction. This technique requires the value of the pressure wave at each grid point, increasing the computational power requirement. In Kaboutari et al. [20], a gelatin phantom was scanned using LPA transducers, and Helmholtz circular coils received MAET signals for each steering angle. However, conductivity images were not reconstructed from MAET signals.

In this study, the imaging performance of MAET‐MI is enhanced by increasing the SNR using a high‐quality factor circular coil, expanding the imaging area coverage with a figure‐of‐eight coil, and improving lateral resolution through the deconvolution of images with the point spread function (PSF). Circuit representation of the air‐cored circular coil is used to derive equations simplifying the estimation of coil parameters. Impedance of the coil is measured over a range of frequencies and circuit parameters of the coil is calculated using this measurement. Then, a method is developed to find the transfer function of a high‐quality factor circular coil by using its measured impedance and calculated circuit parameters. In addition to the circular coil, a circuit representation of the figure‐of‐eight coil is presented and a real figure‐of‐eight coil is made by wounding two high‐quality factor circular coils and connecting them in series. Its circuit parameters are calculated with the same equations derived for a circular coil by using its measured impedance. Then, the transfer function of the figure‐of‐eight coil is estimated using its calculated circuit parameters. Incorporating the transfer function of high‐quality factor circular and figure‐of‐eight coils, the induced MAET signal on the coils is obtained accurately from its output voltage. The noise performance of high‐quality factor coils is compared to coils with a unity quality factor. Significant improvement in SNR was demonstrated using high‐quality factor coils. 16‐element linear phased array (LPA) ultrasound transducer, an air core circular, and a figure‐of‐eight coil are numerically modeled in COMSOL Multiphysics. The LPA Transducer steers the acoustic wave from $-20^{{}^{\circ}}$ to $20^{{}^{\circ}}$ in $1^{{}^{\circ}}$ step angles. The forward problem of MAET is solved for three different two‐dimensional (2D) conductivity distributions. The point spread function is characterized for this imaging modality. It is demonstrated that lateral resolution can be significantly enhanced by performing a 2D deconvolution of the PSF and the 2D sector scan image of the reconstructed conductivity distribution. In addition, the relation between the reciprocal electric field and sensitive regions of the circular coil is shown. For a 2D conductive body, the figure‐of‐eight coil is suggested to be used as a receiver at the locations where the circular coil is insensitive. The conductive boundary images of both coils are brought together to cover the whole domain. Finally, a 2D image of the breast model is obtained. To do this, the model is rotated for 16‐step angles while keeping the LPA transducer steady. At each rotation angle, a sector scan conductive boundary image is obtained for both coils. Then, their lateral resolution is improved by taking 2D deconvolution of the images with their PSF. 2D images are brought together to obtain a complete image.

This study makes several notable contributions to the literature in MAET‐MI: (1) It demonstrates, for the first time, the superiority of high‐quality factor coils over low‐quality factor coils in MAET‐MI, significantly enhancing the Signal‐to‐Noise Ratio (SNR), which is crucial for achieving accurate conductivity reconstructions, particularly in low SNR environments. (2) A practical method is suggested to estimate the coil's transfer function. (3) The induced voltage on the high‐quality factor coil is recovered by deconvolving the estimated transfer function. (4) circuit representation of the figure of eight coils is presented and its transfer function is estimated for the first time in literature (5) Lateral resolution of sector scan conductivity images is improved by deconvolving them with PSF in MAET imaging. (6) Finally, this research is the first to combine images obtained from circular and figure‐of‐eight coils in MAET‐MI imaging to form a comprehensive picture of the entire imaging area.

In the following section of the article, the method's forward problem is mentioned. LPA ultrasound transducer, the circular and figure‐of‐eight coils are numerically modeled. The third section shows the inverse problem solution. In the fourth section, numerical results are shown. Sector scan images of various conductivity distributions are obtained, and the combined use of the circular and figure‐of‐eight coils is demonstrated. Sector scan images are converted from polar to Cartesian coordinate transformation. A 2D image of the breast model is obtained with both coils. The lateral resolution of the image is improved using 2D deconvolution with PSF. The article concludes with a discussion and a conclusion.

## 2 Forward Problem

The forward problem of the MAET method based on magnetic field measurement was previously studied in [19]. When there is conductivity perturbation in the domain, the received signal detected by the receiver coils is expressed as follows:

(1) $$\Delta v_{\mathrm{ind}}=\int_{V_{\text{body}}}{\vec{E}}_{R}\left(\vec{r}\right)\cdot \left(\frac{\partial \vec{v}\left(t\right)}{\partial t}\times \vec{B}\right)\Delta \sigma \left(\vec{r}\right)\mathrm{dv}$$

where $\Delta v_{\mathrm{ind}}$, $V_{\text{body}}$, $E_{R}$, Δσ, $\vec{v}\left(t\right)$ and $\vec{B}$ are the induced voltage on the receiver coil, the integrated volume, the reciprocal electric field of the coil, delta electrical conductivity which is difference between conductivity distribution to homogeneous background conductivity distribution, the particle velocity, and the static magnetic field, respectively.

A high‐quality factor coil is used in this study. Therefore, the effect of its transfer function should be considered. The output of the coil is the multiplication of the transfer function of the coil and the induced signal in the frequency domain:

(2) $$V_{\text{out}}\left(\omega \right)=V_{\mathrm{ind}}\left(\omega \right)\times {\mathrm{TF}}_{\text{coil}}\left(\omega \right)$$

here, $V_{\text{out}}\left(\omega \right)$, $V_{\mathrm{ind}}\left(\omega \right)$, and ${\mathrm{TF}}_{\text{coil}}\left(\omega \right)$ are the output of the coil, induced MAET signal on the coil and the transfer function of the coil in the frequency domain, respectively. When a high‐quality factor coil is used in the experiments, the transfer function of the coil should be found to obtain the induced MAET signal.

In this section, a circuit model of an air‐cored circular coil is shown, and a practical method is suggested to estimate the transfer function of the coil. Noise performance of high and unity quality factor coils is compared. Then, simulations of a numerical model of the LPA Ultrasound transducer, circular, and figure‐of‐eight coils are shown.

### 2.1 Deriving Transfer Function of the Coil

**FIGURE 1:**
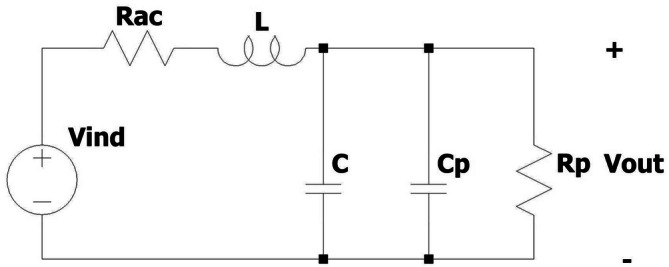
Circuit representation of a circular coil with cable.

In MAET with magnetic field measurements, circular coils are connected to amplifiers via coaxial cables. These cables introduce a parallel capacitance and a resistance. The circuit representation of the circular coil, including a parallel capacitance and resistance, is shown in Figure 1. Here, L, C, and $R_{\mathrm{AC}}$ are the coil's inductance, capacitance, and ac resistance, respectively. $C_{p}$ and $R_{p}$ are the cable's capacitance and resistance, respectively. Relation between $V_{\text{out}}$ and $V_{\mathrm{ind}}$ is as follows Equation (3) [23, 24]:

(3) $$V_{\text{out}}=\frac{Z_{C}//Z_{C_{p}}//R_{p}\times V_{\mathrm{ind}}}{Z_{C}//Z_{C_{p}}//R_{p}+R_{\mathrm{AC}}+Z_{L}}$$

In order to simplify the equation (3), $R_{\mathrm{AC}}+Z_{L}$ and $Z_{C}//Z_{C_{p}}//R_{p}$ are represented as $Z_{1}$ and $Z_{2}$, respectively. Then the transfer function (${\mathrm{TF}}_{\text{coil}}$) can be expressed as:

(4) $${\mathrm{TF}}_{\text{coil}}=\frac{V_{\text{out}}}{V_{\mathrm{ind}}}=\frac{Z_{2}}{Z_{1}+Z_{2}}$$

Note that the total impedance of the coil ($Z_{1}+Z_{2}$) can be measured with an LCR meter over a range of frequencies. Series resistance‐inductance or impedance of the coil without connecting any cable is measured with LCR meter. Then, resonance frequency($f_{r}$) is found at the location of maximum impedance. Then, upper and lower frequencies at which the related impedance takes the impedance value 3 dB below the maximum impedance are found. After that quality factor of the coil Q can be found with [23]:

(5) $$Q=\frac{f_{r}}{\Delta f_{\mathrm{bw}}}$$

here $f_{r}$ is the resonance frequency and $\Delta f_{\mathrm{bw}}$ is the bandwidth of the coil calculated by the upper and lower 3‐dB frequencies of the total impedance. After finding the Q factor, the ac resistance ($R_{\mathrm{ac}}$) of the coil is found with Equation (6) [23].

(6) $$R_{\mathrm{ac}}=\frac{|Z_{\max}\left(2\pi f_{r}\right)|}{Q^{2}}$$

Here, $|Z_{\max}\left(2\pi f_{r}\right)|$ is the absolute value of the maximum impedance of the coil. After finding $R_{\mathrm{ac}}$, L and C can be found using the Equations (7) and (8).

(7) $$L=\frac{{\mathrm{QR}}_{\mathrm{ac}}}{2\pi f_{r}}$$

(8) $$C=\frac{1}{{\mathrm{QR}}_{\mathrm{ac}}2\pi f_{r}}$$

Up to now $Z_{1}$ in Equation (4) is found using estimated $R_{\mathrm{ac}}$ and L. $Z_{1}+Z_{2}$ in Equation (4) is found by measuring the impedance of the coil with the cable at different frequencies with the LCR meter. This cable is used to connect the coil to the amplifier. If the resonance frequency of the coil needs to be tuned to a selected frequency, the tuning capacitor is placed in parallel to the output of the coil and then, cable is connected to the output of the coil. After estimating the circuit parameters of the coil, the transfer function is calculated using Equation (4). Algorithm 1 briefly explains estimation of the coil's transfer function step by step.

ALGORITHM 1 **Estimating Transfer Function of the Coil Using Impedance Measurements With LCR Meter.**

1. Impedance of the coil is measured over different frequencies

2. Maximum impedance, resonance frequency, and bandwidth of the coil are found

3. Q factor of the coil is calculated.

4. $R_{\mathrm{ac}}$ is found using Q factor and $Z_{\max}$

5. L is found using Q factor and $R_{\mathrm{ac}}$

6. Impedance *Z*_1_ is found using *R*_*ac*_ and L.

7. Impedance *Z*_2_ is found by subtracting the measured impedance of the coil, and Impedance *Z*_1_.

8. Transfer function of the coil is estimated by dividing Impedance *Z*_2_ with measured Impedance of the coil.

**FIGURE 2:**
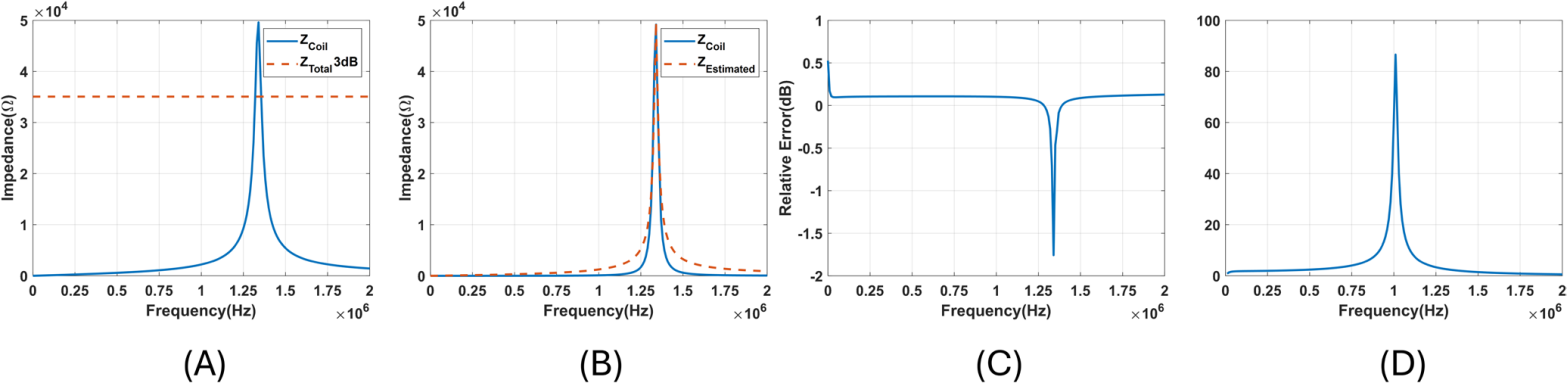
(A) Blue line: Measured impedance of the coil. Orange Dashed Line: The level that is 3 dB lower than the maximum impedance. (B) Blue line: Measured Impedance over Frequency. Orange dashed line: Calculated impedance over frequency. (C) Logarithmic plot of relative error between measured and calculated impedance of the coil. (D) Calculated Transfer function of the coil.

A coil with a 30 mm diameter is wounded with 35 turns of wire with a 0.02 mm diameter. Its impedance over 10 kHz to 2 MHz frequency is measured with an Agilent 4980a LCR meter. Impedance over different frequencies can be seen in Figure 2A. In this plot, the blue line shows the impedance, and the orange dashed line shows the impedance level 3 dB lower than the maximum impedance. The upper and lower 3 dB frequencies are determined by identifying the locations where the orange dashed line intersects with the blue line. The coil's bandwidth is calculated to be 330 kHz. The resonance frequency and the absolute value of maximum impedance are found as 1.34 MHz and 49.6 kΩ. Using these values, Q factor, L, C, $R_{\mathrm{ac}}$ are found as 67, 87.94 μs, 160 pF, and 11.05 Ω. Using these parameters, the measured and calculated impedance over the frequency range are plotted in Figure 2B. The log plot of the relative error between measured and estimated coil impedance is shown in Figure 2C. To find the transfer function of the coil, the impedance of the coil, together with the cable impedance, is measured. Since $Z_{1}$ is already calculated, $Z_{2}$ is calculated by subtracting $Z_{1}$ from the measured impedance of the coil with cable. Then, the transfer function is calculated using equation (4). The calculated transfer function is shown in Figure 2D.

**FIGURE 3:**
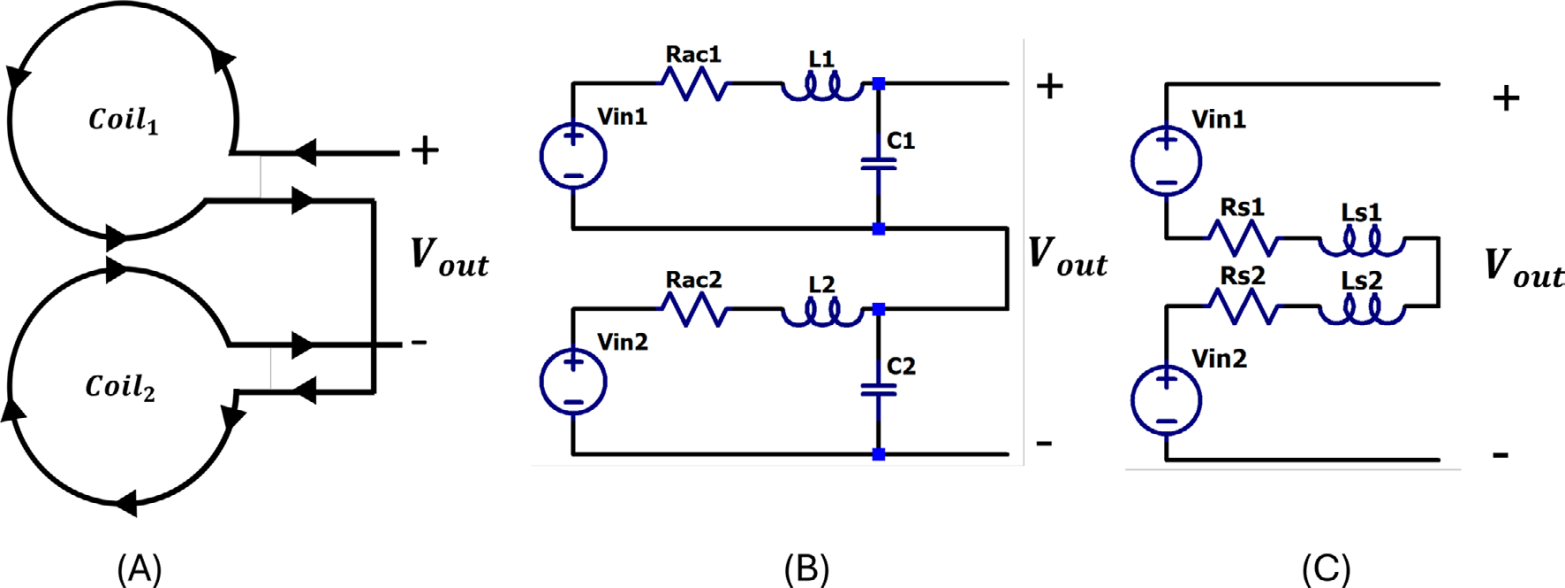
(A) The figure‐of‐eight coil is made up of two circular coils connected in series. The induced current direction is presented with triangles on wires. The current is in the same direction at the junction of the figure‐of‐eight coil. (B) Circuit schematic of the figure‐of‐eight coil. Two circuit representations of circular coils are connected in series. (C) Circuit representation of the figure‐of‐eight coil with series resistance and series inductance.

The figure‐of‐eight coil consists of two circular coils connected in serial as seen in Figure 3A. They are connected such that currents flowing through their wounds are in the same direction at their junction. Circuit representation of the figure‐of‐eight coil is presented with a series connected two circuit schematics of circular coils as seen in Figure 3B. The circuit of the circular coil can be represented by the series resistance (R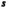) and inductance (L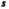) as seen in Figure 3C. Since the two coils are connected in series, the series resistance and inductance of the figure‐of‐eight coil are obtained by summing these values of the individual coils. The figure‐of‐eight coil is realized with connecting two circular coils in series. These circular coils have 35 turns and their diameters are 30 mm. These coils are named as C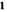 and C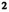. Series resistance and inductance of the circular coils and the figure‐of‐eight coil are measured from 10 kHz to 2 MHz frequency with an Agilent 4980a LCR meter. Using series resistance and inductance, value of circuit components, maximum impedance(Z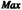), and Quality Factor(Q) of the circular coils and the figure‐of‐eight coil are calculated. In addition to these values, measured series resistance and inductance values are written in Table 1. As shown in this table, the maximum impedance, the series resistance and inductance of the figure‐of‐eight coil are close to the sums of the corresponding values of the circular coils. In contrast, the resonance frequency and Q factor of the figure‐of‐eight coil are approximately similar to those of the circular coils.

**TABLE 1:** Measured series resistance and inductance of the circular coils and figure‐of‐eight coil are shown with *R*_s_ and *L*_s_.

	*R*_s_ (Ω)	*L*_s_ (mH)	*Z*_max_ (kΩ)	*Q*	*R*_ac_ (Ω)	*L* (μH)	*C* (pF)
C_1_ Coil	18,402	2.4	36,173	24.6	59.77	190.27	87.99
C_2_ Coil	14,078	2.4	36,315	24.6	60.09	191.01	87.65
Figure‐of‐eight coil	35,600	5.1	74,835	30.5	80.44	320	53.17

*Note:* *Z*_max_, *Q*, *R*_ac_, *L*, and *C* represent the maximum impedance, quality factor, AC resistance, inductance, and capacitance values calculated from *R*_s_ and *L*_s_.

**FIGURE 4:**
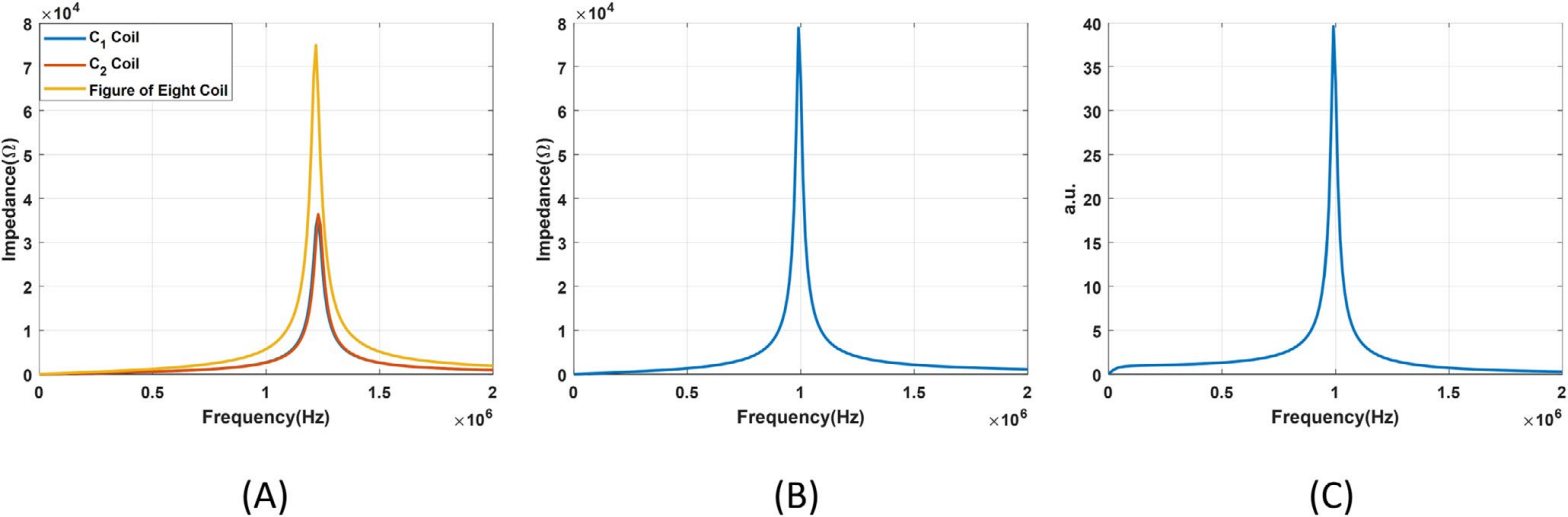
(A) Measured impedance of the figure‐of‐eight, C_1_ and C_2_ coils are presented with yellow, blue and red lines (B) Measured impedance of the figure‐of‐eight coil together with coaxial cable. (C) Estimated transfer function of the figure‐of‐eight coil with coaxial cable.

The measured impedance of the figure of the eight coil and the circular coils can be seen in Figure 4A. In this figure, yellow, red, and blue lines show the impedance of the figure‐of‐eight coil, coil C_1_ and C_2_. To find the transfer function of the figure‐of‐eight coil, impedance of coil together with the coaxial cable and 4.7 pF tuning capacitance are measured with the LCR meter. Measured impedance can be seen in Figure 4B. Transfer function of the figure‐of‐eight coil is estimated using impedance of the coil together with the cable, calculated *R*_ac_ and L components of the coil using equation 3 and 4. Estimated transfer function is seen in Figure 4C.

### 2.2 Noise Performance of High‐Quality Factor Circular and Figure‐of‐Eight Coils

**FIGURE 5:**
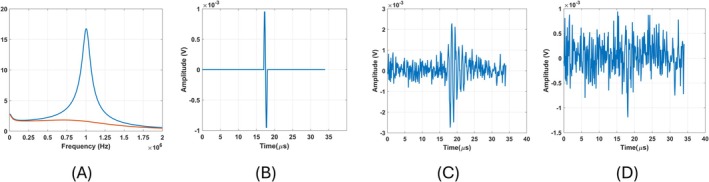
(A) Estimated transfer functions of the high and unity‐quality factor coils. (B) The input signal induced on both high and unity‐quality factor coils (C) The output of the high‐quality factor coils with 3 dB SNR. (D) The output of the unity‐quality factor coil with −9.4 dB SNR.

To show the effect of the quality factor on the noise performance, the quality factor of the coil in the previous section is modified by adding parallel resistances (10 kΩ and 500 Ω). When 10 kΩ and 500 Ω resistances are added, the quality factors of the coil become 10 and 1, respectively. Then, the transfer functions of both coils are estimated and shown in Figure 5A. In this figure, blue and orange lines show the transfer functions of coils with 10 and 1, respectively. To compare the noise performance of these coils, a single cycle sinusoidal signal with a frequency of 1 MHz (Figure 5B) is applied to both coils. The noise level that produces a 3 dB SNR for the high‐quality factor coil is added to the coil's output with a unity quality factor. The output voltages for both coils are shown in Figure 5C,D. While the high‐quality factor coil has an SNR of 3 dB, the unity‐quality factor coil has an SNR of −9.4 dB. This indicates that the high‐quality factor coil improves the SNR by 12.4 dB in the same noisy environment.

**FIGURE 6:**
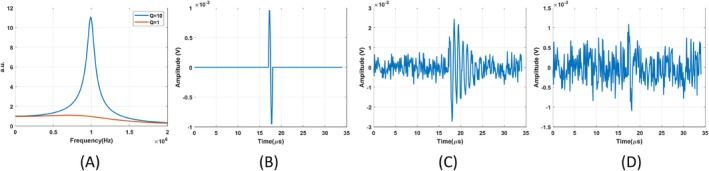
(A) Estimated transfer functions of the high and unity‐quality factor of the figure‐of‐eight coil. (B) The input signal induced on both the high and unity‐quality factor of the figure‐of‐eight coil. (C) The output of the high‐quality factor of the figure‐of‐eight coil with 3 dB SNR. (D) The output of the unity‐quality factor coil with −9.59 dB SNR.

The quality factor of the figure‐of‐eight coil is adjusted to 10 and 1 by adding 40 and 2 kΩ resistors in parallel, respectively. Transfer function of these coils can be seen in Figure 6. Blue and orange lines in this figure show transfer function of the figure‐of‐eight coil with quality factor of 10 and 1, respectively. A single cycle sinusoidal signal with a frequency of 1 MHz (Figure 6B) is applied to both coils. Noise is introduced to the output of the figure‐of‐eight coil with a higher quality factor, resulting in an output signal‐to‐noise ratio (SNR) of 3 dB. Resulting signal can be seen in Figure 6C. Same noise is introduced to the output of the figure‐of‐eight coil with a lower quality factor. The output signal can be seen in Figure 6D. The signal‐to‐noise ratio (SNR) of this signal is −9.59 dB. This observation indicates that the high‐quality factor of the figure‐of‐eight coil enhances the SNR by 12.59 dB under the same noisy conditions. The SNR improvements resulting from higher quality factors of both the circular coil and the figure‐of‐eight coil are comparable. This outcome is expected, as noise performance is determined by the quality factor of the coils.

### 2.3 LPA Ultrasound Transducer Modeling

**FIGURE 7:**
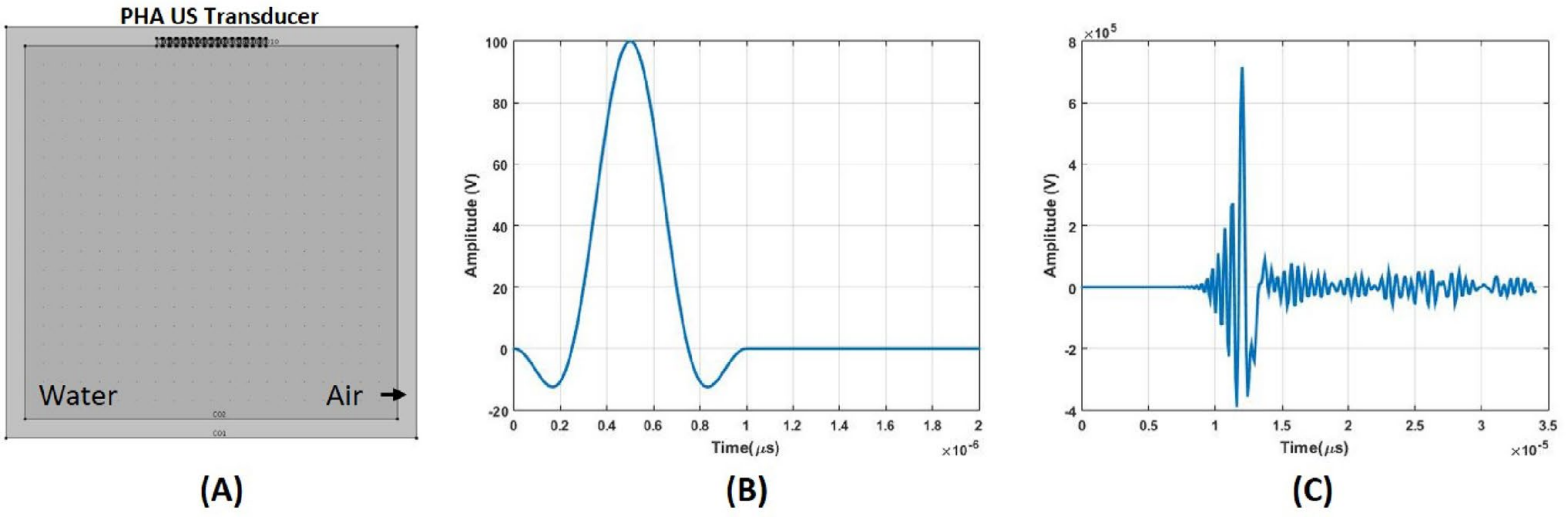
(A) Image of 2D numerical model and LPA transducer. (B) The excitation signal for each element of the LPA transducer. (C) The particle velocity at 20 mm away from the transducer.

**FIGURE 8:**
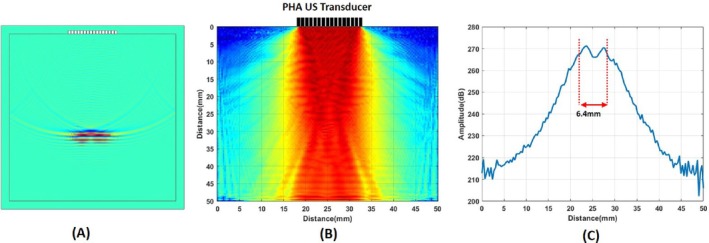
Logarithmic image of ultrasound intensity produced by LPA transducer for 0° steering angle. (B) Lateral profile of the ultrasound intensity produced by LPA transducer. 3 dB beamwidth is shown with red dashed lines.

Linear phased array (LPA) ultrasound transducer with 16‐elements is modeled in COMSOL Multiphysics [25]. The material of each element is PZT‐5H piezoelectric crystals. The width of elements is 0.66 mm, and the spacing between two neighboring elements is 0.33 mm. The transducer is placed at the top of the imaging domain as shown in Figure 7A. The speed of sound and density of the domain were chosen as 1520 m/s and 1000 kg/m^3^, respectively. The imaging domain is surrounded by air (speed of sound 326 m/s). The size of the imaging domain is 50 mm by 50 mm divided into 149 by 149 elements. The mesh size of the imaging domain is chosen as 0.33 mm, which is one‐fifth of the acoustic wave's wavelength. The elements of the LPA transducer are driven by a one‐period sine wave with an amplitude of 100 V, a frequency of 1 MHz, and a phase of 270°. This excitation signal is windowed using a Tukey window [26]. The Tukey window is applied in practical excitations of ultrasound transducers [20]. The use of a 270° signal phase combined with a Tukey window concentrates energy on the positive side of the wave. By concentrating energy on the positive side, it becomes easier to observe changes in the phase of the MAET signals at the transitions between regions of higher to lower and lower to higher conductivity. The applied electric potential and particle velocity at a center point are shown in Figure 7B,C, respectively. The transducer steers packed acoustic wave from −20° to 20° 1° step angles. Particle velocity waveform for 0° steering angle at COMSOL simulation can be seen in Figure 8A. The intensity pattern of the LPA transducer is drawn in Figure 8B. The lateral cut is taken at the center and drawn in Figure 8. −3 dB beamwidth of the LPA transducer is found to be 6.4 mm.

### 2.4 Computation of the Reciprocal Electric Field of Circular and Figure‐Of‐Eight Coils

**FIGURE 9:**
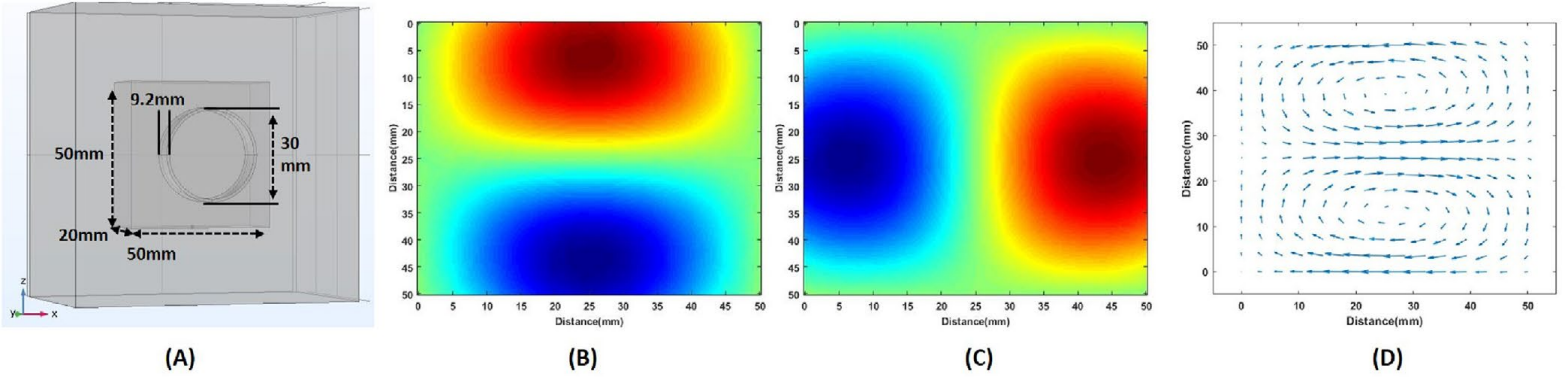
(A) Circular coil modeled in COMSOL. (B) The reciprocal field is generated by a circular coil in the direction of x. (C) The reciprocal field is generated by a circular coil in the direction of z (D) Arrow plot of the reciprocal field of a circular coil.

The reciprocal electric field of a coil is defined as the electric field generated within the body when a time‐varying current of 1 A is applied to the coil [27]. In this study, air‐cored circular coils and figure‐of‐eight coils are modeled in COMSOL Multiphysics. The thickness, inner diameter, and outer diameter of a 35‐turn circular coil are 30 mm, 31 mm, and 9.2 mm, respectively. The reciprocal electric field of the coil is calculated in rectangular prism whose dimensions are 50 × 50 × 20 mm, as shown in Figure 9A. The coil is placed 2 mm away from the rectangular prism, and its center is aligned with its center. The coil is excited with a 1 MHz current having 1 A magnitude. x and z‐component of the reciprocal electric field on the slice 10 mm below the surface are shown in Figure 9B,C, respectively. In Figure 9D, The arrow plot shows the x and z components of the circular coil's reciprocal electric field.

**FIGURE 10:**
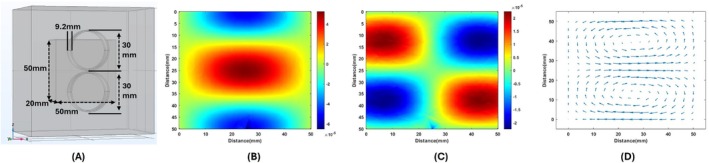
Figure‐of‐eight coils. COMSOL model (A). X component of the reciprocal field (B). Z component of the reciprocal field (C). Arrow plot of the reciprocal field (D).

Figure 9B,C clearly show that the reciprocal electric field of the circular coil is very low near the center. This means that it is insensitive to conductivity variation in this region. To address this issue, the figure‐of‐eight coil is investigated for its potential use in the MAET‐MI imaging method. The figure‐of‐eight coils are two circular coils placed on top of each other. They are connected using opposite winding directions. Because of this reason, the reciprocal electric field of both coils is added up at the junction points. Therefore, they are sensitive to the junction point of two coils. This feature makes them suitable to use as stimulator coils in transcranial magnetic stimulation devices to stimulate smaller regions of the brain [28, 29]. Also, the figure‐of‐eight coil configuration can be used to obtain a signal where the circular coil is not sensitive for the same reason. To investigate this, the figure‐of‐eight coil is modeled in Comsol as seen in Figure 10A. Two 35‐turn‐circular coils having a thickness of 9.2 mm, an inner diameter of 30 mm, and an outer diameter of 31 mm are placed in a row. It is placed 2 mm in front of a rectangular prism having 50 × 50 × 20 mm size. The conductivity of a rectangular prism is 1 S/m. X and Z components of electric fields at the center cross‐section area are drawn in Figure 10B,C. The arrow plot of the electric field is shown in Figure 10D.

## 3 Inverse Problem

High‐quality factor coils are used in this study. As seen in Equation (2), the output of the coil is the multiplication of the transfer function of the coil and the induced signal on the coil. The induced signal on the coil is obtained from the output of the coil to reconstruct conductive boundaries. For this purpose, induced signals on the coil are reconstructed by deconvolving the output signal with the transfer function of the coil by using Wiener Filter [30, 31] as presented below:

(9) $$V_{\mathrm{ind}}\left(\omega \right)=\frac{V_{\text{coil}}\left(\omega \right)\times {\mathrm{TF}}^{*}\left(\omega \right)}{{\left|\mathrm{TF}\left(\omega \right)\right|}^{2}+\epsilon }$$

Here, $V_{\mathrm{ind}}\left(\omega \right)$, $V_{\text{coil}}\left(\omega \right)$, $\mathrm{TF}\left(\omega \right)$ and ϵ represent the Fourier Transforms of induced voltage on the coil, the output voltage of the coil, transfer function of the coil and an empirical constant related to the ratio of the spectral density of noise, respectively. Given the difficulty in determining ϵ, a common approximation is to use $0.01{\left|\mathrm{TF}\left(\omega \right)\right|}_{\max}^{2}$ as ϵ [30]. The induced signal obtained through the Wiener filter is utilized in solving the inverse problem. To solve the inverse problem, the forward problem of MAET is reformulated as the convolution of the pressure wave and conductivity, following the approach described by Zhou et al. [17]. In this work, an acoustic pressure wave is applied through the conductive material under a static magnetic field, and the resulting current is measured with electrodes. A similar conversion is performed for MAET‐MI in our previous work [21]. Here, the forward problem of MAET was reformulated as the convolution of the conductivity and time derivative of the particle velocity with a scalar multiplication of the reciprocal electric field for the 0° steering angle. Wiener filter is used in deconvolution to reconstruct conductivity distribution. This approach is used in this study. The magnetic field in the forward problem of MAET‐MI seen in Equation (1) is assumed to be constant and directed solely in the y‐direction, then Equation (1) transforms into [21]:

(10) $$\Delta v_{\mathrm{ind}}\left(t\right)=B_{y}\int_{V}\left(\frac{\partial v_{x}\left(\vec{r},t\right)}{\partial t}E_{z}\left(\vec{r}\right)-\frac{\partial v_{z}\left(\vec{r},t\right)}{\partial t}E_{x}\left(\vec{r}\right)\right)\Delta \sigma \left(\vec{r}\right)\mathrm{dr}$$

The LPA ultrasound transducer steers the ultrasound wave in the z direction with a limited span. The majority of the acoustic energy is concentrated in the $\vec{v}z$ component. Consequently, the term $\frac{\partial v_{x}\left(\vec{r},t\right)}{\partial t}E_{z}\left(\vec{r}\right)$ in the equation is omitted. Given that the particle velocity is a confined wave propagating in the z‐direction, The influence of the conductivity perturbation in the y‐direction is neglected. Thus, Equation (10) simplifies to the following form in Cartesian coordinates [21]:

(11) $$\Delta v_{\mathrm{ind}}\left(t\right)=-B_{y}\int_{x}\int_{z}\frac{\partial v_{z}\left(x,t-z/c\right)}{\partial t}E_{x}\left(x,z\right)\Delta \sigma \left(x,z\right)\text{dxdz}$$

Limit of integral in z direction is determined by length of the imaging domain and limit of x is determined by beamwidth of the pressure wave. Since the particle velocity is propagating in the z‐direction and conductivity variation is considered in z direction, the integral in the x‐direction remains constant. $\tau =\frac{z}{c}$ and $\mathrm{d\tau}=\frac{\mathrm{dz}}{c}$ is substituted in Equation (11), Also, $h\left(\tau \right)$, and $u\left(t-\tau \right)$ are substituted with $E_{x}\left(\tau \right)\Delta \sigma \left(\tau \right)$ and $\frac{\partial v_{z}\left(t-\tau \right)}{\partial t}$ in Equation (11), the forward problem becomes a convolution in the z‐direction [21]:

(12) $$\Delta v_{\mathrm{ind}}\left(t\right)=C\int_{l_{z}}u\left(t-\tau \right)h\left(\tau \right)\mathrm{d\tau}$$

here, $u\left(t-\tau \right)$, $h\left(\tau \right)$, and $l_{z}$ represents time derivative of particle velocity in z‐direction, scalar multiplication of delta conductivity multiplied with x component of reciprocal electric field and the distance traveled by the propagating acoustic wave, and C is a constant that incorporates $B_{y}$ and the integral in the x‐direction. $h\left(\tau \right)$ can be found using Wiener Filter [30, 31] by deconvolving the time derivative of particle velocity and induced MAET signal on the coil as shown below:

(13) $$H\left(\omega \right)=\frac{V_{\text{coil}}\left(\omega \right)\times U^{*}\left(\omega \right)}{{\left|U\left(\omega \right)\right|}^{2}+\;\epsilon }$$

here, $H\left(\omega \right)$, $V_{\mathrm{ind}}\left(\omega \right)$, $U\left(\omega \right)$ and ϵ represent the Fourier Transforms of $h\left(z\right)$, the voltage induced on the coil, the time derivative of particle velocity and empirical constant related to the ratio of the spectral density of noise to that of the signal respectively. By performing the inverse Fourier transform of the $H\left(\omega \right)$, $h\left(z\right)$ is obtained. Since $h\left(z\right)$ is $E_{x}\left(\tau \right)\Delta \sigma \left(\tau \right)$, Delta conductivity scaled by the reciprocal electric field is obtained. The rest of the article, h(z), is reconstructed.

### 3.1 Transition From Polar to Cartesian Coordinates

**FIGURE 11:**
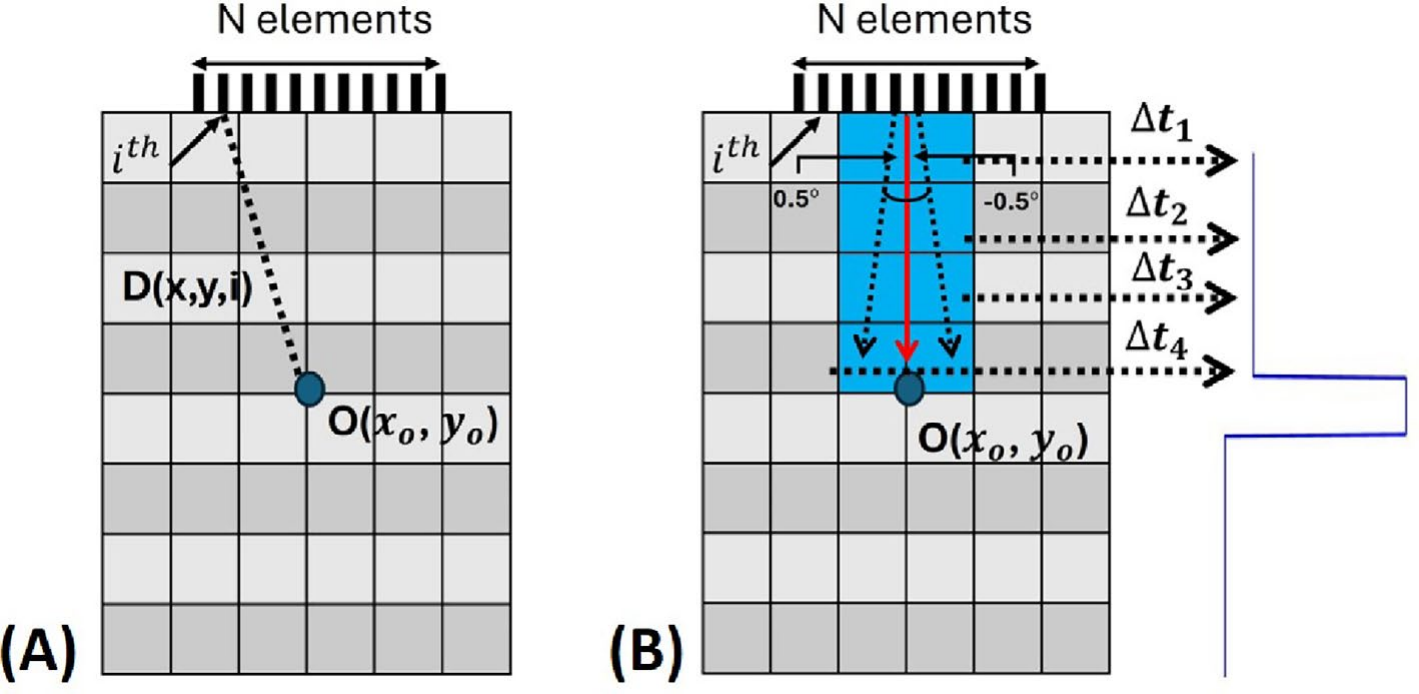
(A) Illustration of delay‐and‐sum algorithm. (B) Blue line shows the example conductivity distribution, red arrow shows the steering direction and dashed black arrows are 0.5° higher and lower than the steering angle.

In this study, acoustic waves are steered in 1° step angles in 40° span. The MAET voltage induced on the coil is calculated at each steering angle. Conductive boundaries are reconstructed at each angle. In this way, sector scan images are obtained, like ultrasound imaging. These images are polar coordinate images, and they can be converted to cartesian coordinates using the delay‐and‐sum Method (DAS) in ultrasound imaging. In this method, the echo signals received by each element are delayed and summed according to their position in the array [32]. Then, delays are applied to the echo signal of each element according to the steering angle. Then, these echo signals are summed. The imaging area is divided into pixels. The distance of each pixel to the transducer element is found as seen in Figure 11. The duration of time passed between the pixel and transducer element is found by dividing the distance by the speed of sound. Then, the value of the summed signals at the corresponding duration of time is assigned to the value of the corresponding pixel as shown in Equation (15). Since pixels having the same distance to the element of the transducer take the same value, artifacts occur on the image. All pixels with the same distance to elements are not selected to decrease these artifacts. Instead, pixels are selected between 0.5° more and less than the scan angle. For 0° steering angle, the selected pixels are blue in Figure 11B. Duration of time values are then calculated according to the distances of these pixels to the center point of the transducer. According to this calculation, the pixels are assigned the corresponding reconstructed conductivity value as seen in Figure 11B. In this manner, pixels within a 40° range are populated.

(14) $$\Delta t=\frac{D\left(x,y\right)\times 2}{c}$$

(15) $$I\left(x,y\right)=\sum_{i=0}^{N}S\left(i,\Delta t\left(x,y,i\right)\right)$$

## 4 Numserical Results

### 4.1 Point Spread Function for MAET‐MI

**FIGURE 12:**
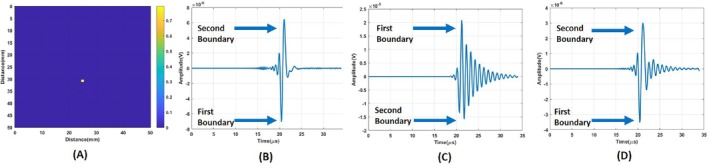
(A) Blood tissue positioned 33.5 mm away from the transducer. (B) MAET signal induced on the circular coil. (C) Output of the coil after the MAET signal was induced. (D) Recovered MAET signal by deconvolving the transfer function of the coil.

The point spread function is found using the smallest detectable object. The wavelength of a single‐period sinusoidal pressure wave is 1.5 mm at 1 MHz frequency. Hence, the size of the object to be imaged is determined as slightly bigger than that. Regarding the mesh size, the object's size was selected as 1.65 mm. In the imaging area, the conductivity distribution consists of two layers: breast fat (0.025 S/m) and blood tissue (0.822 S/m) region [33, 34, 35]. The length and width of the imaging domain is 50 × 50 mm. The size of blood tissue is 1.65 × 1.65 mm, respectively. Blood tissue seen as a yellow square in Figure 12A is placed 33.5 mm away from the transducer. A homogeneous magnetic field of 1 T was applied in the y‐direction. The MAET signal induced in the circular coil for the blood tissue is depicted in Figure 12B. MAET signals resulting from Lorentz currents induced on the first and second boundaries of the blood tissue are shown with arrows in Figure 12B.

The output of the coil after the MAET signal is induced on the coil is the convolution of the Transfer function of the coil. It is shown in Figure 12C. The estimated transfer function seen in Figure 2D is deconvolved from the output of the coil using the Wiener filter. The resulting induced signal is plotted in Figure 12D.

**FIGURE 13:**
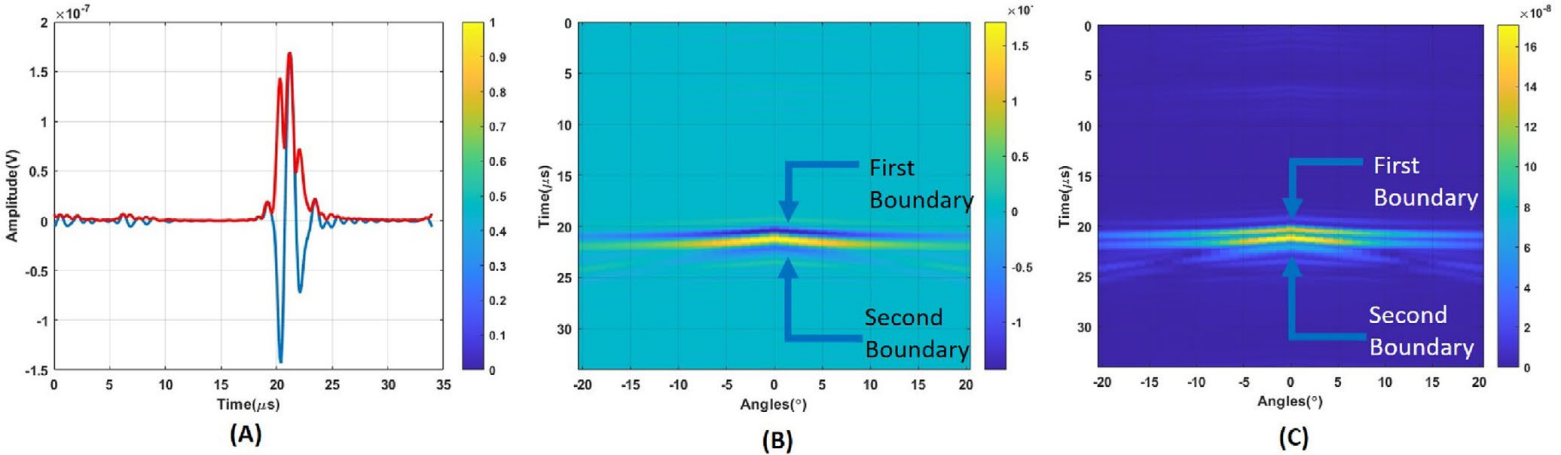
(A) The Wiener Filter Results for 0° steering angle. (B) Reconstructed conductive boundaries of all angles for the circular coil. (C) Envelope of Reconstructed conductive boundaries image.

In Equation (13), Fourier Transform of the time derivative of the particle velocity at center seen in Figure 7C and reconstructed induced MAET signal seen in Figure 12D are used for $U\left(\omega \right)$ and $V\left(\omega \right)$, respectively. The result of Equation (13) can be seen as a blue solid line in Figure 12A. The MAET‐MI method is based on packed sinusoidal acoustic waves, the total length of which is shorter than the length of the conductivity variation. As a result, the integral in Equation (1) yields non‐zero values only at the boundaries of conductivity variations. This implies that the Wiener filter identifies the locations of the boundaries between regions with different conductivities. To obtain a smoother image, the envelope of the conductive boundary image is taken as shown with a red line in Figure 13A. LPA ultrasound transducer steers ultrasound wave from −20° to 20° with 1° step angles. The same process is applied to MAET signals at each steering angle. Reconstructed conductive boundaries are placed column by column 2D image as shown in Figure 13B. Envelopes of the reconstructed conductive boundaries are plotted in Figure 13C. As a point spread function, a Reconstructed conductive boundary image of conductivity distribution seen in Figure 13B is used for images obtained using the circular coil.

**FIGURE 14:**
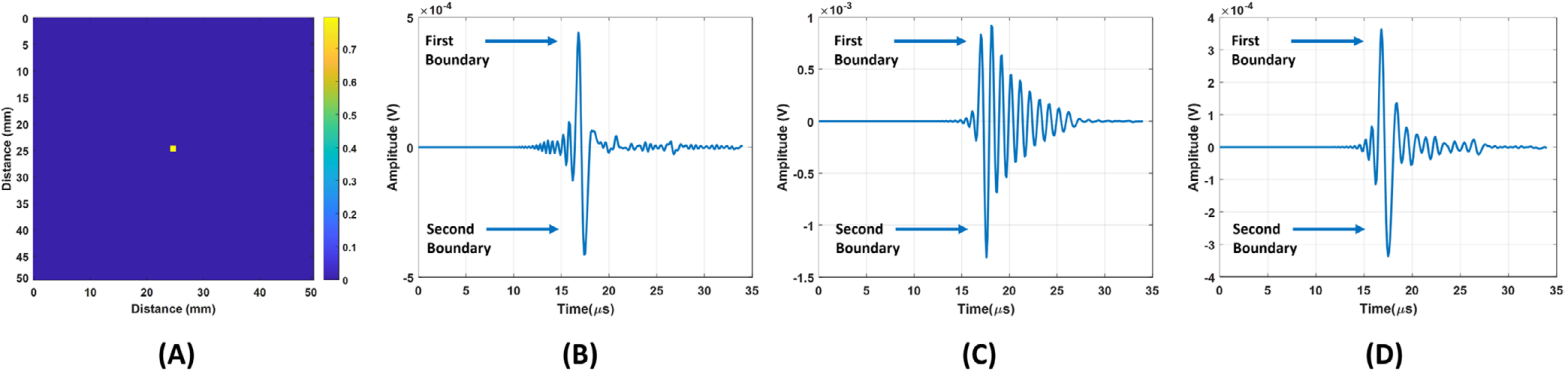
(A) Blood tissue positioned 25 mm away from the transducer. (B) MAET signal induced on the figure‐of‐eight coil. (C) Output of the figure‐of‐eight coil after MAET signal induced. (D) Recovered MAET signal by deconvolving the transfer function of the coil.

For the figure‐of‐eight coil, blood tissue is placed at the center as seen in Figure 14A. The MAET signal induced on the figure‐of‐eight coil for the blood tissue is depicted in Figure 14B. MAET signals resulting from Lorentz currents induced on the first and second boundaries of the blood tissue are shown with arrows in Figure 14B. The output of the figure‐of‐eight coil for a quality factor of 10 is shown in Figure 14C. The estimated transfer function seen in Figure 6A is deconvolved from the output of the coil using the Wiener filter. The resulting induced signal is plotted in Figure 14D.

**FIGURE 15:**
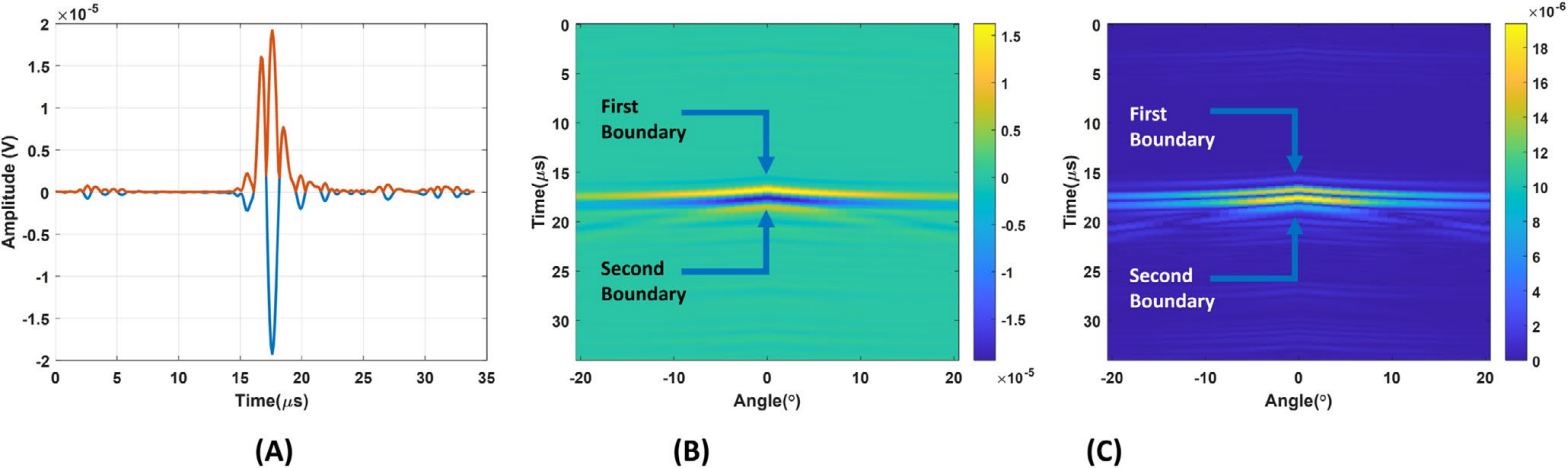
(A) The Wiener Filter result for 0° steering angle (blue line) and envelope of the Wiener Filter result(red line). (B) Reconstructed conductive boundaries of all angles for the figure‐of‐eight coil. (C) Envelope of Reconstructed conductive boundaries image.

Conductivity boundaries are reconstructed using recovered MAET signal induced on the figure‐of‐eight coil and time derivative of particle velocity using the Wiener filter. Result of the Wiener filter and envelope of the result can be seen in Figure 15A as blue and red, respectively. As in the circular coil case, LPA ultrasound transducer steers ultrasound waves and MAET signals are obtained at each angle. Reconstructed conductivity boundaries and their envelopes are seen in Figure 15B,C. Reconstructed conductive boundary image of conductivity distribution seen in Figure 15B is used for images obtained using the figure‐of‐eight coil.

**FIGURE 16:**
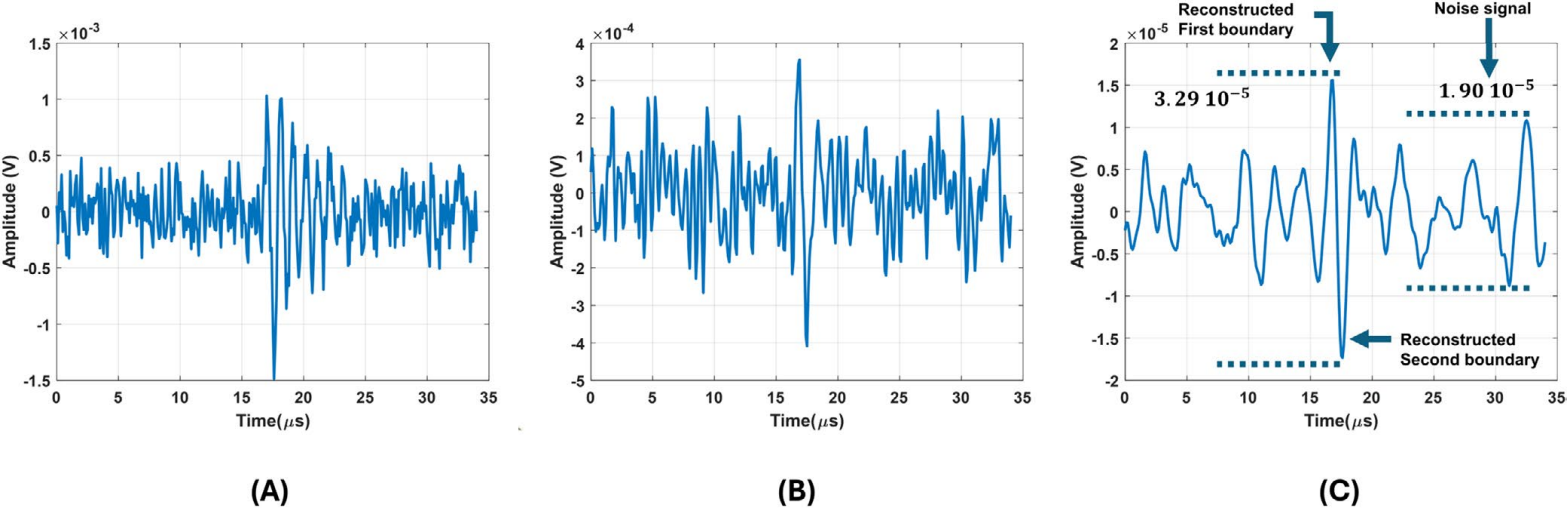
(A) Output of the figure‐of‐eight coil after MAET signal induced for 0 dB SNR. (B) Recovered MAET signal by deconvolving the transfer function of the figure‐of‐eight coil. (C) Reconstructed conductive boundaries for 0 dB SNR of the output signal of the figure‐of‐eight coil.

**FIGURE 17:**
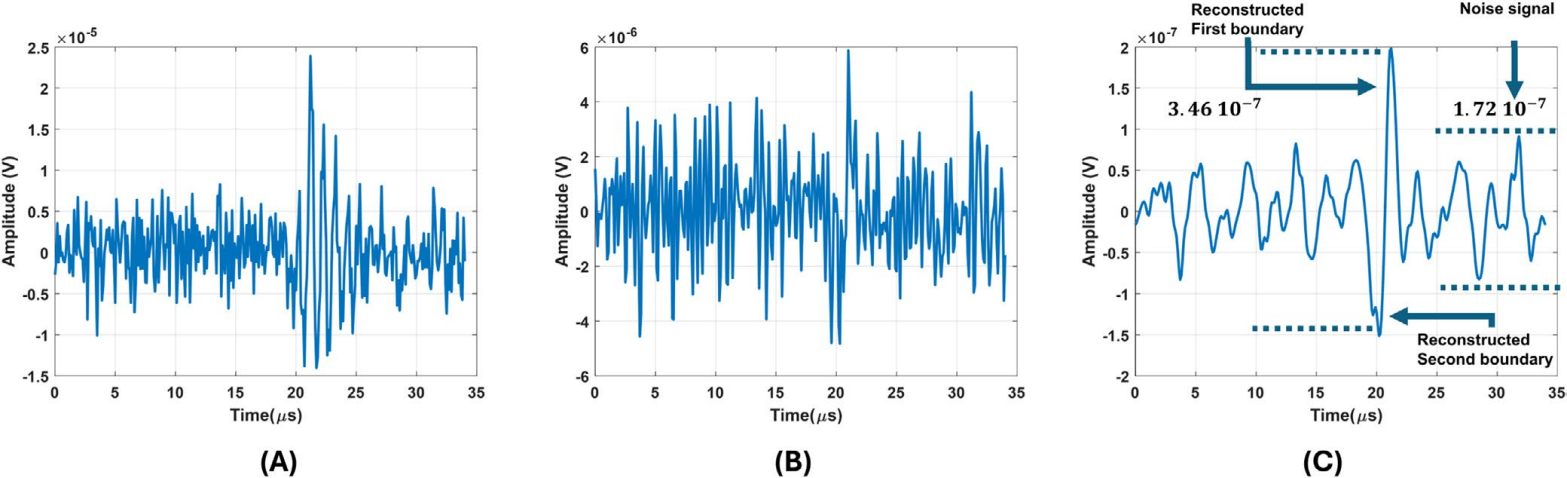
(A) Output of the circular coil after MAET signal induced for 0 dB SNR. (B) Recovered MAET signal by deconvolving the transfer function of the circular coil. (C) Reconstructed conductive boundaries for 0 dB SNR of the output signal of the circular coil.

Noise tolerance of the reconstruction algorithm is tested by adding noise to the output of the circular and figure‐of‐eight coil and reconstructing conductivity boundaries. The added noise is enough to make the SNR of the output signal of the figure‐of‐eight coil 0 dB, as seen in Figure 16A. The recovered induced signal using the Transfer function of the figure‐of‐eight coil can be seen in Figure 16B. Corresponding reconstructed conductivity boundaries can be seen in Figure 16C. In this figure, the amplitude of reconstructed conductivity boundaries is at least 3 dB larger than the highest noise amplitude. This amplitude difference gets below 3 dB when SNR is adjusted below 0 dB. Therefore, 0 dB is the minimum SNR that conductivity boundaries can be distinguished from noise. For the circular coil, the output signal having 0 dB SNR can be seen in Figure 17.

### 4.2 Evaluation of Sensitivity of Circular and Figure‐Of‐Eight Coils

**FIGURE 18:**
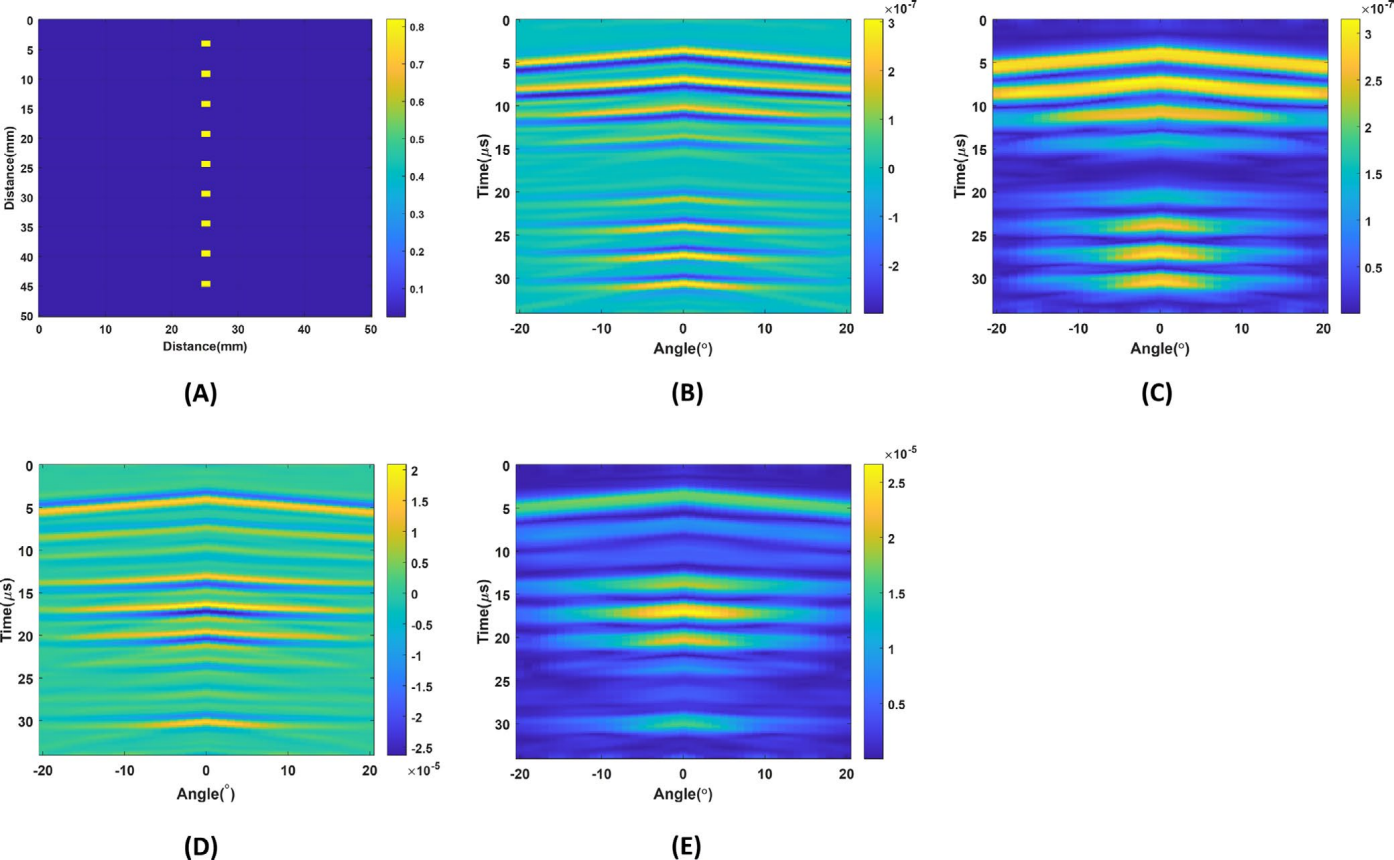
(A) 2D conductivity distribution of nine blood tissues. (B) Reconstructed conductive boundary image obtained with the circular coil. (C) Envelope of the reconstructed conductive boundary image obtained with the circular coil. (D) Reconstructed conductive boundary image obtained with the figure‐of‐eight coil. (E) Envelope of the reconstructed conductive boundary image obtained with the figure‐of‐eight coil.

The circular coil's reciprocal electric field is shown in Figure 9D. To evaluate its sensitivity, nine blood tissues having 1.65 mm size are placed in the region of interest, as given in Figure 18A. These objects are blood tissues having a conductivity value of 0.82 S/m at 1 MHz. The objects are placed in a fat tissue background with 0.025 S/m conductivity. Ultrasound is placed on top of the region of interest. Each element of the LPA transducer is excited with a 100 V amplitude and 270° phase added to a single cycle 1 MHz Tukey windowed sinusoidal signal. The LPA transducer steers ultrasound waves from −20° to 20° with a 1° step angle. The resulting MAET signals are deconvolved with the time derivative of particle velocity using a Wiener filter at each angle. The resulting 2D conductive boundary image is seen in Figure 18B. The envelope of the conductive boundary image is shown in Figure 18C.

In the region where the electric field takes higher values, the stronger MAET signals are received. MAET signals get lower where the electric field strength gets close to zero. The reciprocal electric field is very low near the center of the coil. As seen in Figure 18B,C, MAET signals diminish near the coils' center. Also, as seen in these figures, blood regions have elongated tails. The reason is that the LPA transducer steers the parallel wave, and the beamwidth of the parallel wave is large. Since the beamwidth of the LPA transducer intensity pattern is 6.4 mm, even a 1.65 mm size object appears larger than −3 dB beamwidth in the reconstructed image.

When the figure‐of‐eight‐coils are used to obtain MAET signals from the same conductivity distribution in Figure 18A, 2D reconstructed conductive boundary image can be seen in Figure 18D. Envelope of the reconstructed conductive boundary image can be seen in Figure 18E. As shown in these figures, blood tissues located 10 mm and 25 mm below the LPA transducer are not reconstructed due to the proximity of the center of the figure‐of‐eight coil to these regions.

#### 4.2.1 Improving Lateral Resolution Using PSF

**FIGURE 19:**
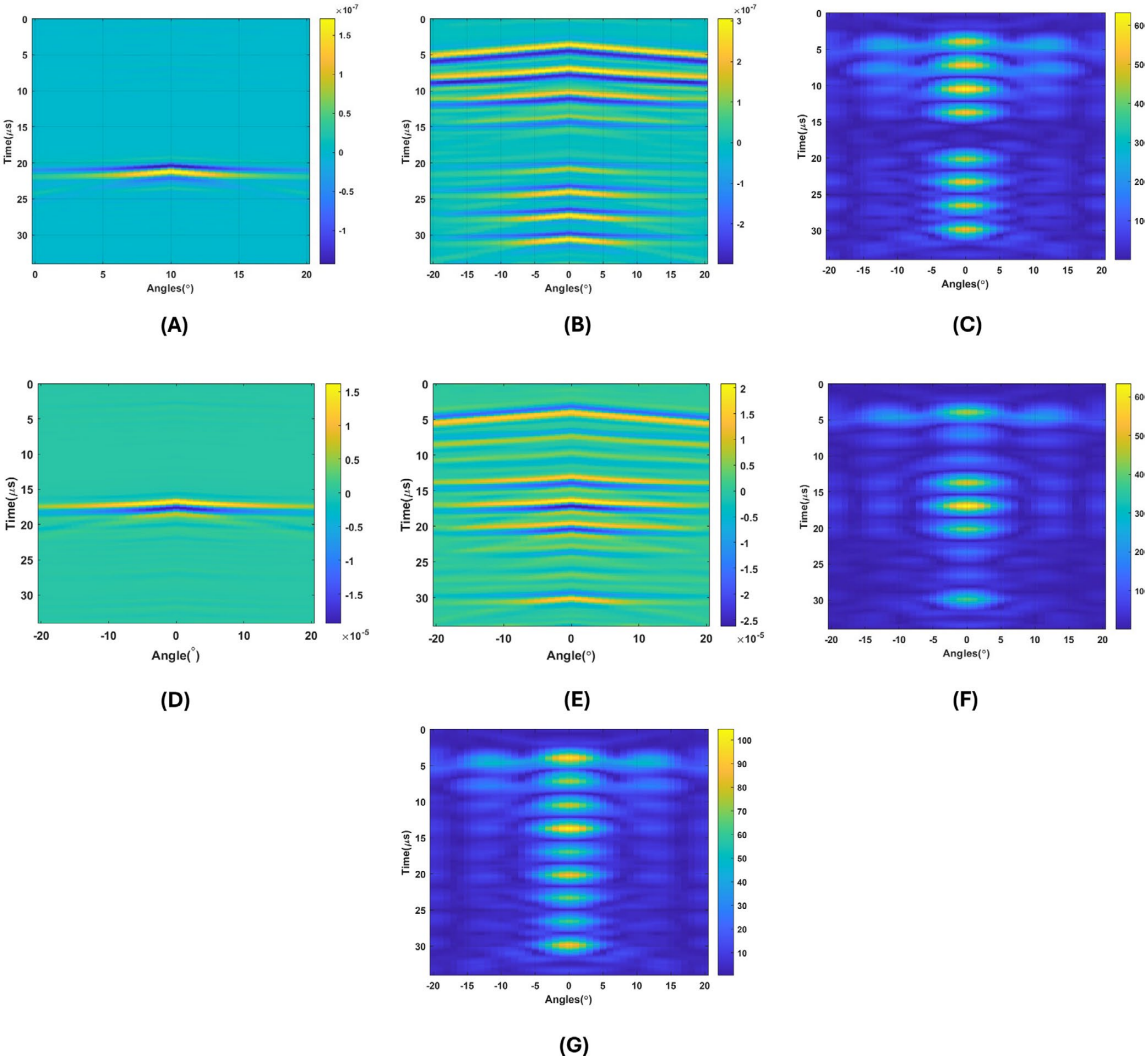
(A) 2D conductive boundary image of point spread function for circular coil case. (B) 2D Reconstructed conductive boundary image of 9 blood tissues for the circular coil. (C) Result of the 2D deconvolution of conductive boundary image with PSF. (D) 2D conductive boundary image of point spread function for figure‐of‐eight coil case. (E) 2D Reconstructed conductive boundary image of 9 blood tissues for the figure‐of‐eight coil. (F) Result of the 2D deconvolution of conductive boundary image with PSF.(G) Summation of envelope of the 2D deconvolution of conductive boundaries with PSF obtained by the circular and figure‐of‐eight coils.

Lateral resolution can be enhanced by performing a 2D deconvolution of the image and the point spread function (PSF), as commonly implemented in ultrasound imaging [36]. The reconstructed conductive boundary image shown in Figure 19B is deconvolved using the PSF corresponding to the circular coil, as illustrated in Figure 19A. The envelope of the resulting image is then extracted and presented in Figure 19C, demonstrating a significant reduction in the long tails on either side of the objects, thereby improving lateral resolution. The same deconvolution process is applied to the reconstructed conductive boundary image obtained with the figure‐of‐eight coil, depicted in Figure 19E. This image is deconvolved using the PSF associated with the figure‐of‐eight coil, as shown in Figure 19D. The resulting envelope is extracted and displayed in Figure 19G. The widths of the blood tissues in the images deconvolved with the point spread function and those without deconvolution have been compared. The edges of the blood tissue are taken as half of the maximum index. The widths of the blood tissues cover 16° in reconstructed conductivity images without deconvolution of PSF depicted in Figure 18C,E. On the other hand, they cover 8° in reconstructed conductivity images with deconvolution of PSF depicted in Figure 19C,F. This shows deconvolving the reconstructed conductivity images with PSF improves the lateral resolution of the images.

#### 4.2.2 Combining Images of Circular and Figure‐Of‐Eight Coil

The figure‐of‐eight and circular coils exhibit sensitivity to different regions within the same domain, as shown in Figure 19C,F. Therefore, these two images are combined to achieve coverage of the entire domain. The resulting image is presented in Figure 19G, demonstrating that all blood tissues are successfully covered and imaged.

### 4.3 Two‐Dimensional Conductive Boundary Imaging

**FIGURE 20:**
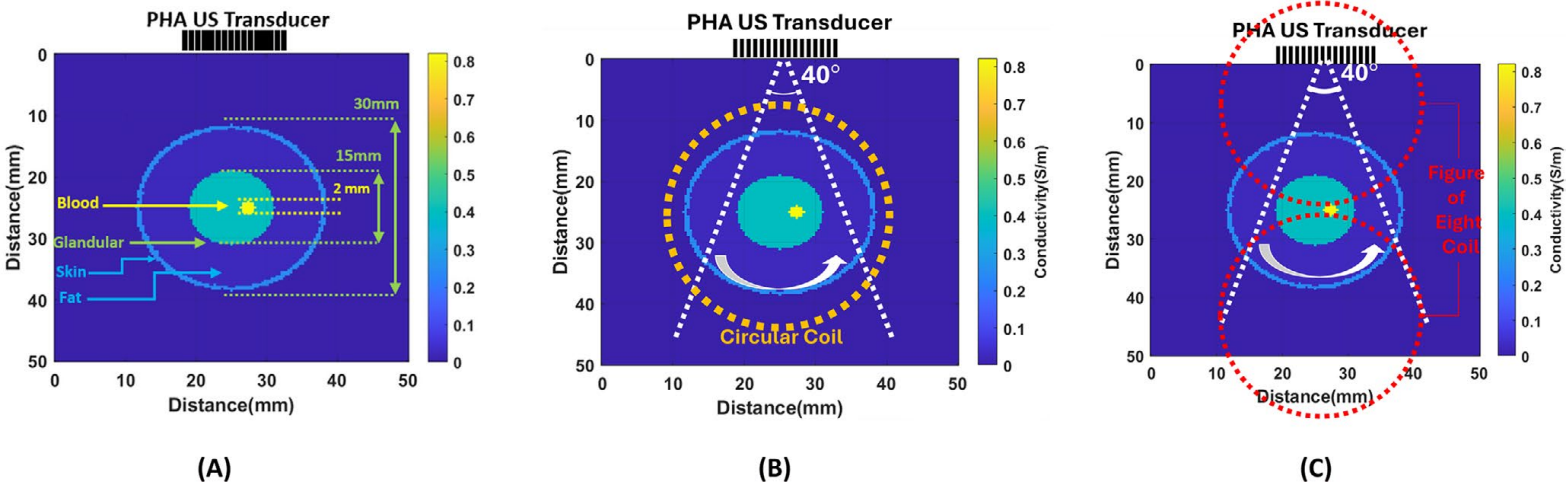
(A) Dimension of tissues in the original conductivity distribution (B) Location of a circular coil and the limit angles of sector scanning in an imaging domain. (C) Location of a figure‐of‐eight coils on the original conductivity distribution.

The imaging area is rotated in front of the LPA ultrasound transducer to achieve high‐resolution images. As the LPA transducer scans each sector, the conductivity distribution is rotated by a step angle. The resulting sector scan images are then converted to Cartesian coordinates and combined. To evaluate this method, the simplified breast model shown in Figure 20A is used. This conductivity distribution consists of four parts: skin tissue (0.025 S/m), breast fat (0.025 S/m), breast gland (0.4 S/m), blood tissue (0.822 S/m), and water (0 S/m). The skin tissue is represented by a green circle with a diameter of 26.4 mm. The blood tissue appears as a red‐filled circle with a 2 mm diameter. The breast gland tissue appears as a light green‐filled circle with a 5.3 mm diameter. Fat tissue is located between the skin and the breast gland. Outside the skin, tissue is represented as water, depicted in navy blue in Figure 20B. The breast model is rotated counterclockwise in 16 steps, each with a step angle of 22.5°. At each angle, an LPA transducer is placed on top of the conductivity distribution and directs the pressure wave from −20° to 20° in 41 steps. The range of sectors is shown by the dashed white lines in Figure 20B. Once the induced signals have been recorded on the receiver coil, the tissues are rotated at a step angle. The LPA transducer then scans the sector. The location of the circular and figure‐of‐eight coils on the conductivity distribution can be seen by the orange and red dashed circles in Figure 20B,C, respectively. MAET signal data acquisition and conductivity boundary reconstruction algorithm are summarized in Algorithm 2.

ALGORITHM 2 **MAET Signal Data Acquisition and Conductivity Boundary Reconstruction Algorithm.**

1. LPA Ultrasound Transducer applies pressure wave to the body at a selected steering angle.

2. Output of the coil is recorded during the acquisition period.

3. MAET signal induced on the coil is recovered by deconvolving the output of the coil and the impulse response of the coil.

4. Conductive boundaries are reconstructed by deconvolving the recovered MAET signal with the time derivative of the particle velocity.

5. 1D Conductive boundaries are interpolated on the slice corresponding to the selected angle.

6. The process from the first bullet point to the fifth bullet point is repeated for the subsequent steering angles until a total span angle of 40° is completed.

7. Resolution of the conductive boundary image is increased by taking 2D deconvolution with the PSF image and the reconstructed conductive boundary image.

8. The breast model is rotated by a specified rotation step angle.

9. The process from the first bullet point to the seventh bullet point is repeated until all 16 rotation angles are completed.

**FIGURE 21:**
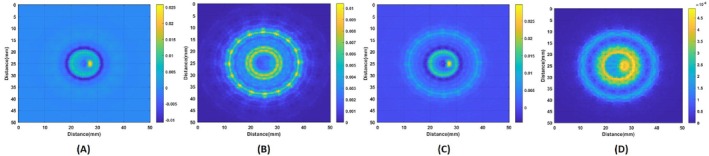
(A) Reconstructed conductive boundary image obtained using the figure‐of‐eight coil and (B) circular coil. (C) Reconstructed conductive boundary image by summing images from both coils. (D) Reconstructed conductive boundary image without 2D deconvolution of PSF.

The 2D deconvolution of the conductive boundary image with the PSF is performed at each rotation of the conductivity distribution. These images are then mapped to Cartesian coordinates according to their rotation angle. Once 16 images are obtained, the complete image of the object is reconstructed. The conductive boundary images deconvolved with PSF for the figure‐of‐eight coil and the circular coil are shown in Figure 21A,B, respectively. As illustrated, the figure‐of‐eight coil and the circular coil are sensitive to different regions of the imaging domain. By combining the two images, a complete image of the conductivity distribution can be obtained, as seen in Figure 21C. The reconstructed conductive boundary image without 2D deconvolution with PSF is presented in Figure 21D. As illustrated in Figure 21D, it is clearly observable that the blood, tissue, and skin are integrated with one another. The tissues are also larger than the original ones. Conversely, the image with deconvolution exhibits enhanced resolution, allowing for the individual identification of tissues. This demonstrates the impact of deconvolution with PSF on the image resolution.

**FIGURE 22:**
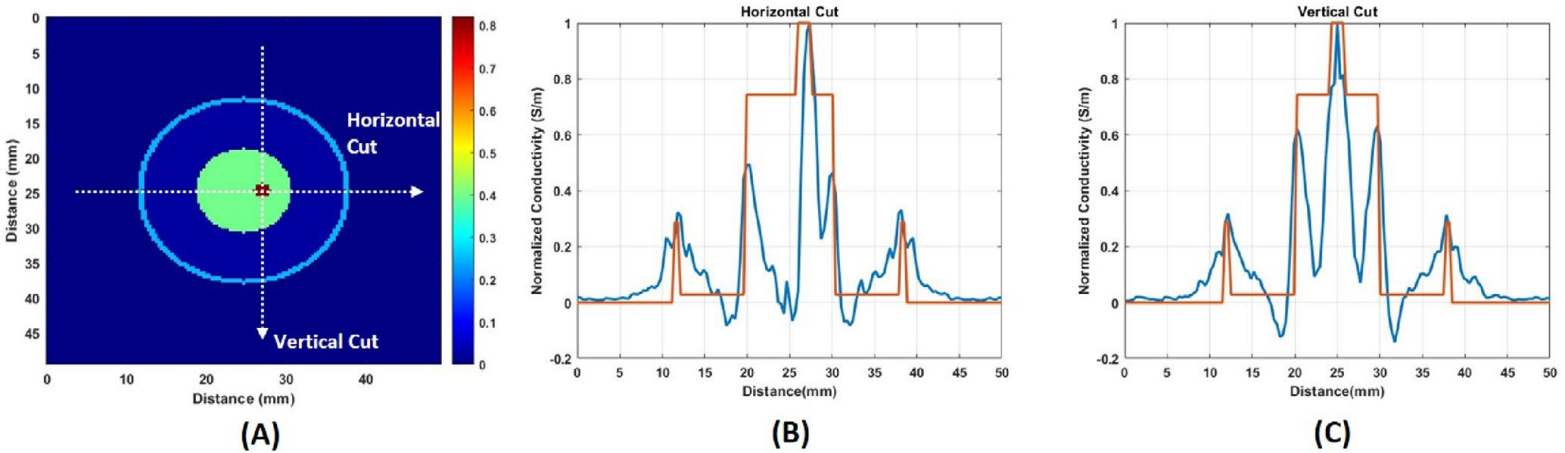
(A) Original conductivity distribution and location of the horizontal and vertical cuts. (B) Horizontal and (C) vertical cuts that pass through the center of the blood tissue.

To facilitate a comparison between the original conductivity distribution and the reconstructed conductive boundary images, horizontal and vertical cuts are taken through the center of the blood tissue, as illustrated in Figure 22A. The normalized plots of these vertical and horizontal cuts for both the original and reconstructed images are displayed in Figure 22B,C, respectively (Table 1).

## 5 Discussion

In this study, we examine the impact of a high‐quality factor circular coil on the imaging performance of Magneto‐Acoustic Electrical Tomography with Magnetic field measurements (MAET‐MI). Our findings highlight the superiority of high‐quality factor coils over their lower‐quality counterparts. Specifically, the comparison of noise performance shows that high‐quality factor coils enhance the signal‐to‐noise ratio (SNR) by approximately 12.4 dB, a significant improvement essential for effective signal reconstruction. Additionally, the sharper bandpass characteristics of these coils make them suitable for single‐frequency applications. However, their low damping ratio affects the signal, which we address by deconvolving the coil's transfer function from its output. We propose a practical method for estimating the coil's transfer function by calculating circuit parameters from measured impedance over a range of frequencies. In addition to the circular coil, a circuit representation of the figure‐of‐eight coil is drawn and its circuit parameters are calculated from its measured impedance over a range of frequencies. The coil transfer function of the figure‐of‐eight coil is estimated from its calculated circuit parameters for the first time in the literature.

Numerical studies are conducted to find the reciprocal electric field of the coils and particle velocity generated by LPA ultrasound transducer within the imaging domain. We utilized a phased array ultrasound transducer to steer ultrasound waves and acquire MAET signals from sector scans. Conductive boundaries are reconstructed by deconvolving the time derivative of the particle velocity from the induced voltage. The reconstruction of the conductive boundaries using the Wiener Filter presents a viable approach to solving the inverse problem in MAET. While the LPA transducer steers ultrasound waves, the reconstructed conductive boundaries are placed at each angle. This method results in the creation of sector scan conductive boundary images. The relation between the sensitivity map of an air‐core circular coil and its reciprocal electric field pattern is revealed. We showed that circular coils are not responsive to conductivity changes near their center but are highly sensitive to areas beneath their windings. To cover the entire domain effectively, a figure‐of‐eight coil is recommended to be used in conjunction with circular coils. This approach is advantageous because the figure‐of‐eight coil is highly sensitive to the regions under their junction points. Aligning the center of the circular coil with the junction of the figure‐of‐eight coil ensures complete domain coverage.

In this study, we also rotated the breast model in 16 steps, with the phased array transducer performing sector scanning at each rotation angle. The received MAET signals were processed to obtain conductive boundary images, which were then converted to Cartesian coordinates based on their rotation angle and summed. This process yielded comprehensive conductive boundary images for both circular and figure‐of‐eight coils, as these coils are sensitive to different regions. Summing these images provided a complete representation. The phased array transducer's sector scanning method, due to its high beamwidth pressure wave, reduces lateral resolution. For the first time in this imaging modality's literature, the resolution of sector scan conductive boundary images has been enhanced. The Point Spread Function (PSF) for this modality is defined and performs 2D deconvolution of sector scan images at each rotation using the PSF. After summing the resulting images, it is clearly demonstrated that higher resolution is achieved through the use of PSF.

## 6 Conclusions

This study makes significant contributions to the literature on Magneto‐Acoustic Electrical Tomography (MAET) with magnetic field measurements. It demonstrates the advantage of high‐quality factor coils over lower‐quality factor coils by showing a corresponding improvement in the signal‐to‐noise ratio (SNR) of the induced signal. A practical method is proposed for estimating the circuit parameters and transfer function of a circular coil by measuring its impedance across a range of frequencies. In addition to the circular coil, a circuit model of a figure‐of‐eight coil is introduced. The circuit parameters and transfer function of the figure‐of‐eight coil are estimated based on its measured impedance using an LCR meter. To the best of our knowledge, this is the first time in the literature that the transfer function of a figure‐of‐eight coil has been estimated from its calculated circuit parameters.

Additionally, the reciprocal electric fields of circular and figure‐of‐eight coils are simulated using COMSOL. An ultrasound transducer is modeled in COMSOL, and the particle velocity distribution is analyzed for different steering angles. The forward problem is solved at each angle, and the MAET signals induced on the circular and figure‐of‐eight coils are determined for various conductivity distributions. Wiener filtering identifies conductive boundaries by deconvolving particle velocity and MAET signals at each angle. This approach significantly reduces the processing power needed for conductivity reconstructions using particle velocity measurements at a single point.

Furthermore, the sensitivity map of circular coils is demonstrated. For a two‐dimensional body, the figure‐of‐eight coil is suggested for use alongside circular coils to achieve a complete image. A sector scan image of the conductive boundaries in a two‐dimensional simplified breast model is obtained from 16 rotation angles. The images obtained using circular and figure‐of‐eight coils are merged to produce a comprehensive image. Merging conductive boundary images obtained using two different types of coils is the first in this imaging technique.

Despite the low resolution resulting from the large beamwidth of the LPA transducer, the study introduces a point spread function for this imaging modality to enhance resolution. Each sector scan conductive boundary image is deconvolved with the point spread function, resulting in a notable increase in resolution.

Overall, this study advances MAET‐MI by enhancing imaging performance through improved SNR with a high‐quality factor circular coil, expanded coverage with a figure‐of‐eight coil, and better lateral resolution via a deconvolving image with PSF, offering significant contributions to the field.

## Conflicts of Interest

The authors declare no conflicts of interest.

## Data Availability

The data that supports the findings of this study are available in the supplementary material of this article.
